# Backbone diversity beats text supervision: a systematic study of frozen multi-foundation model fusion for in-the-wild plant disease recognition

**DOI:** 10.3389/fpls.2026.1860665

**Published:** 2026-08-11

**Authors:** Hajar El Karch, Youssef Natij, Mohamed Benaly, Rachid El Gouri, Abdelkader Mezouari

**Affiliations:** 1 Laboratory of Advanced Systems Engineering, National School of Applied Engineering, Ibn Tofail University, Kenitra, Morocco; 2 Scientific Research and Innovation Laboratory, Higher School of Technology, Ibn Tofail University, Kenitra, Morocco; 3 Innovative Systems Engineering Laboratory, National School of Applied Sciences of Tetouan, Abdelmalek Essaadi University, Tetouan, Morocco

**Keywords:** feature fusion, foundation models, in-the-wild benchmarks, plant disease recognition, PlantDoc, PlantWild, prototypical classification, self-supervised vision transformers

## Abstract

Automated plant disease recognition from field photographs remains challenging: models trained on laboratory datasets collapse on in-the-wild images, and the current state of the art on PlantWild—the largest open in-the-wild benchmark (18,542 images, 89 classes)—relies on text prototypes derived from a language model to reach 76.18% top 1 accuracy. We ask whether text supervision is truly necessary or whether the bottleneck is the *diversity* of the visual representation. Our central methodological finding is that *backbone selection and fusion matter far more than classifier-head engineering*: across 118 experiments, a simple fixed-weight linear–prototype combination on top of three complementary frozen backbones yields larger and more reproducible gains than any head-level adaptive routing mechanism we test. Specifically, through a systematic study of six frozen vision backbones (three CLIP, two DINOv3, and one DINOv2), three classifier heads, and four fusion configurations, completed in a single day on one consumer GPU, we establish three findings. (i) A single self-supervised backbone (DINOv2 ViT-L/14) already surpasses text-augmented MVPDR (77.56% vs. 76.18%). (ii) Concatenating three complementary backbones (DINOv2 + DINOv3 + CLIP) reaches 80.23% ± 0.41% (five seeds), exceeding the *published* MVPDR accuracy by +4.05 points and our *own reproduction* of MVPDR under an identical evaluation protocol by +7.96 points, without any language supervision. (The difference between the two deltas reflects evaluation–protocol differences—our split, model-selection criterion, and training schedule—rather than any discrepancy in the reported numbers; see Section 4.6.6 for a full reconciliation.) (iii) Every form of learned routing we test—per-class gating, backbone gating, sample-wise gating—is inessential; the gain is entirely attributable to backbone diversity and a simple linear–prototype scoring combination. On the smaller PlantDoc benchmark, the same principle transfers but with substantially higher seed variance: the best configuration reaches 80.09% at a favourable seed but 76.97% ± 1.48% over five seeds—a suggestive rather than robust gain. Beyond the accuracy headline, we provide a pathology-aware per-class analysis showing that DINOv2/v3 dominate on fine-texture lesion classes (rusts, mildews, and leaf spots) whilst CLIP’s narrow advantage concentrates on organ/species-level identification (rice leaf and potato late blight). All primary claims are validated over five seeds, and all code, feature caches, and result files are released for full reproducibility.

## 1 Introduction

Crop diseases account for roughly 20%–40% of yearly global yield losses, and image-based recognition of disease symptoms is a foundational component of modern artificial intelligence (AI)-driven plant pathology (Savary et al., 2019). A decade of work on this problem has shown that convolutional networks fine-tuned on PlantVillage (Hughes and Salathé, 2015; Mohanty et al., 2016) (a curated dataset of 54,000+ controlled, single-leaf images spanning 38 disease classes across 14 crop species) trivially reach 99% top 1 accuracy on these controlled images (Ferentinos, 2018; Too et al., 2019), yet the same models collapse to 30%–50% on in-the-wild photographs where occlusions, variable lighting, cluttered backgrounds, and species-specific symptom diversity are the norm (Barbedo, 2018; Noyan, 2022). Closing this laboratory-to-field accuracy gap is the central methodological challenge that the present paper addresses. Throughout, we use “in-the-wild” in the sense used by the benchmark creators (Wei et al., 2024b; Singh et al., 2020)—uncurated images sourced from the internet with realistic acquisition variability—rather than as a synonym for controlled field-deployment capture.

The community responded with two in-the-wild benchmarks that target this distribution gap: PlantDoc (Singh et al., 2020), a 2,598-image multi-crop dataset, and PlantWild (Wei et al., 2024b), the largest open benchmark to date with 18,542 images spanning 89 disease classes. On PlantWild, the MVPDR method (Wei et al., 2024a) currently holds the state of the art at 76.18% top 1: it freezes CLIP ViT-L/14, clusters image features into visual prototypes, enriches them with text prototypes derived from GPT-3.5 class descriptions, and scores test images against the union. We caution that this published figure is not directly comparable to numbers obtained under a different evaluation protocol: when we reproduce MVPDR within our own pipeline (official PlantWild split, identical model-selection and training schedule), we obtain 72.27% ± 0.42%—a 3.91-point gap attributable to protocol differences rather than any error in the original work (see Section 4.6.6 for full details). This result implicitly encodes an assumption widely held in the field: that language supervision is indispensable for closing the lab-to-field recognition gap.

We test the converse hypothesis. Modern self-supervised vision backbones—DINOv2 (Oquab et al., 2024) and DINOv3 (Siméoni et al., 2026)—are trained on hundreds of millions of unlabelled web images and learn dense, locally discriminative features without any text alignment. Their representations are complementary to those of CLIP: DINO captures fine texture, lesion morphology, and part structure; CLIP captures species-level semantics and organ identity. If the in-the-wild gap is a feature-diversity problem rather than a text-supervision problem, then simply combining these frozen representations should close the gap—and text supervision may not be the load-bearing factor for closed-set accuracy.

This paper reports a systematic empirical investigation that confirms this thesis. We contribute the following:

1. Systematic backbone×head study. We evaluate three heads (linear probe, prototype head, and hybrid linear–prototype) on six frozen backbones spanning two pretraining paradigms, on both PlantWild and PlantDoc—36 supervised configurations plus 15 few-shot runs, producing, to our knowledge, the first side-by-side comparison of self-supervised and contrastive backbones on in-the-wild plant disease recognition under a unified protocol.
2. Backbone diversity beats text supervision. Concatenative fusion of DINOv2, DINOv3, and CLIP at the ViT-L scale reaches 80.23% ± 0.41% top 1 on PlantWild (five seeds)—+4.05 pts over published MVPDR (76.18%) and +7.96 pts over our reproduction of MVPDR under an identical protocol (72.27% ± 0.42%)—without any language supervision.
3. Head complexity is inessential. An exhaustive ablation of three routing mechanisms3. (per-class gate, backbone gate, and sample-wise gate) and two initialisation strategies conclusively shows that no form of learned routing contributes once features are diverse; a fixed 0.5/0.5 linear–prototype mixture matches or exceeds every adaptive variant. We frame this as a positive methodological finding: the field can stop over-engineering classifier heads and focus on feature selection.
4. Dataset-size guidance. A stratified subsample sweep on PlantWild at budgets of 25 full images/class shows the three-backbone hybrid head leading everywhere without crossover. On PlantDoc, the head ranking inverts at a single seed but not across five seeds; we recommend the three-backbone hybrid recipe as a robust default.
5. Few-shot evaluation. Backbone choice dominates head choice in the few-shot regime. DINOv2 with a prototype head already reaches 33.80% at *k* = 1—+15.6 points over the best CLIP configuration at the same *k*. Three-backbone fusion reaches 68.07% at *k* = 16, outperforming the CLIP hybrid by +9.87 points and MVPDR by +≈14 points.

We deliberately frame the contribution as an empirical discovery combined with a critical methodological audit, rather than as a new architectural module. We argue that the field has been over-fitting to head-level novelty when a careful examination of which visual representations to combine yields larger and more reproducible gains. All 118 experiments were completed in a single day on one consumer GPU (RTX 3070, 8GB), and all code, cached features, and JSON result files will be released for full reproducibility.

## 2 Related work

### 2.1 Plant disease recognition under distribution shift

Early CNN-based plant disease work (Mohanty et al., 2016; Ferentinos, 2018) achieved very high accuracy on PlantVillage and its variants, but cross-dataset evaluation (Barbedo, 2018) exposed the dependence on background and lighting cues. Noyan (2022) showed that an image-only background classifier reaches ≥99% on PlantVillage, confirming that much of the reported accuracy reflects spurious correlations. PlantDoc (Singh et al., 2020) and PlantWild (Wei et al., 2024b) were designed precisely to break those shortcuts; both are photographer-curated and multi-crop, and exhibit the long-tailed class distributions typical of agricultural field reporting.

### 2.2 Foundation models for visual recognition

CLIP (Radford et al., 2021) demonstrated that contrastive image–language pretraining yields strong zero-shot transfer. The DINO family (Caron et al., 2021; Oquab et al., 2024; Siméoni et al., 2026) showed that self-distillation on unlabelled images produces dense features that excel at part discovery, segmentation, and fine-grained classification. These two families are known to capture different aspects of an image: CLIP encodes semantics that co-occur with text, and DINO encodes texture and local structure (Kornblith et al., 2019). Several recent studies in generic visual recognition observe that fusing self-supervised and contrastive features yields gains over either alone (Zhao et al., 2024). Most closely related in the plant-pathology domain, PlantCaFo (Yang et al., 2025) combines CLIP, DINO, and DINOv2 with a learned adapter and a weight-decomposed fusion module for few-shot leaf disease recognition. Our work differs in three important respects: (i) we keep the fusion strictly non-parametric (plain feature concatenation), which allows us to isolate the diversity effect from any adapter contribution; (ii) we evaluate systematically across six backbones and three heads rather than a single frozen configuration; and (iii) our negative result on learned routing—spanning per-class, per-backbone and per-sample gates—directly challenges the underlying assumption that adapter-style fusion modules are necessary.

### 2.3 Adapting frozen backbones

Linear probing (He et al., 2022) and prototypical heads (Snell et al., 2017) are standard. CLIP-Adapter (Gao et al., 2023) and Tip-Adapter (Zhang et al., 2022) introduce lightweight adapters that combine learned and zero-shot signals. Visual prompt tuning (Jia et al., 2022) and LoRA (Hu et al., 2022) keep the backbone frozen whilst injecting low-rank trainable parameters. We adopt the strictest variant of this paradigm: backbones are completely frozen, all features are pre-extracted to disk, and only a small head is trained.

### 2.4 Multimodal plant disease recognition

MVPDR (Wei et al., 2024a) is the closest baseline: it freezes CLIP ViT-L/14, encodes class names through CLIP’s text encoder to form text prototypes, clusters image features per class to form visual prototypes, and produces logits as a similarity-weighted combination. On PlantWild, they report 76.18% top 1, and on PlantDoc, they report 77.23%. Our work asks whether the text component is load-bearing or whether comparable accuracy can be obtained from a richer purely visual representation.

## 3 Experimental protocol

### 3.1 Datasets

PlantWild (Wei et al., 2024b) is the largest open in-the-wild plant disease benchmark, containing 18,542 images across 89 disease classes drawn from internet sources, with substantial class imbalance (largest class >500 images, smallest <30) and full visual variability (multiple leaves, fruit, soil backgrounds, varying illumination). We use the official train/test split (14,865 train, 3,677 test) and carve a stratified 10% validation slice from the training set using seed = 0 (1,820 val, 13,045 train), held fixed across all experiments. Class-wise counts of the val slice match the original training distribution to within one sample/class.

PlantDoc (Singh et al., 2020) contains 2,598 images across 27 plant disease classes from 13 species, manually photographed in fields and gardens. We use the official train/test split (2,330 train/268 test), yielding approximately 90 training images per class on average.

We do not use PlantVillage (Hughes and Salathé, 2015) for training, given its known background biases (Noyan, 2022) that do not transfer to field conditions.

### 3.2 Frozen backbones

We extract a single global feature vector per image from the [CLS] token (or global-averagepooled patch tokens for ResNets) of six pretrained backbones, listed with their feature dimensions in **Table 1**. Features are extracted once per (backbone, dataset) pair and cached to disk; all subsequent experiments operate on the same cached features, eliminating any source of variance from the backbone.

**Table 1:** Frozen backbones used in this study.

Backbone	Pretraining	Dim.	Input res.
CLIP ResNet-101 CLIP ViT-B/16 CLIP ViT-L/14 DINOv2 ViT-L/14 DINOv3 ViT-L/16 DINOv3 ViT-Huge+	Image–text contrastive Image–text contrastive Image–text contrastive Self-supervised distillation Self-supervised distillation Self-supervised distillation	512 512 768 1,024 1,024 1,280	224 × 224 224 × 224 224 × 224 518 × 518 518 × 518 518 × 518

All backbones are loaded from public checkpoints and completely frozen. DINOv3 (Siméoni et al., 2026) is a peer-reviewed TMLR 2026 publication; checkpoints are available at https://github.com/facebookresearch/dinov3.

### 3.3 Classifier heads

Given a feature vector **x** ∈ R*^D^* and *C* classes, we compare three heads of increasing complexity.

#### 3.3.1 Linear probe

A single fully connected layer Linear(*D,C*) trained with cross-entropy, providing a lower bound and a control for representation quality.

#### 3.3.2 Prototype head

Following the visual-prototype component of Wei et al. (2024a) adapted to a no-text setting, we maintain *K* learnable visual prototypes $\left\{p_{c,k}\right\}\underset{k=1}{K}$ per class, *ℓ*_2_-normalise both inputs and prototypes, and compute (**Equations 1**–**3**).

(1) $${\text{logit}}_{c}\left(x\right)=\frac{1}{K\tau }\sum_{k=1}^{K}\frac{x^{⊤}p_{c,k}}{∥x∥∥p_{c,k}∥},$$

with a learnable temperature *τ* initialised at 0.07. Prototypes are initialised by per-class *k-*means (MacQueen, 1967) on training features.

#### 3.3.3 Hybrid head (linear+prototype)

This head combines a linear path and a prototype path with a fixed equal-weight mixture:

(2) $${\text{logit}}_{c}\left(x\right)=\frac{1}{2}\left(w_{c}^{⊤}\tilde{x}+b_{c}\right)+\frac{1}{2}\frac{1}{K\tau }\sum_{k=1}^{K}{\tilde{x}}^{⊤}{\tilde{p}}_{c,k},$$

with $\tilde{\cdot }$ denoting *ℓ*_2_ normalisation along the feature axis. The linear weights are initialised to per-class means of normalised features, and the prototypes are initialised by per-class *k*-means. We initially hypothesised that a learnable per-class gate *σ*(*α_c_*) should modulate the mixture; our ablation (Section 4.3) conclusively shows that it is unnecessary, so we report all results with the fixed 0.5/0.5 split.

### 3.4 Multi-backbone feature fusion

For a set of *B* backbones with feature dimensions *D*_1_*,…,D_B_*, we concatenate their pre-extracted features:

(3) $${\text{x}}_{\text{fuse}}=\left[{\text{x}}_{1}∥{\text{x}}_{2}∥∥\cdots ∥{\text{x}}_{B}\right]\in {\mathbb{R}}^{\sum_{i}D_{i}.}$$

**Figure 1:**
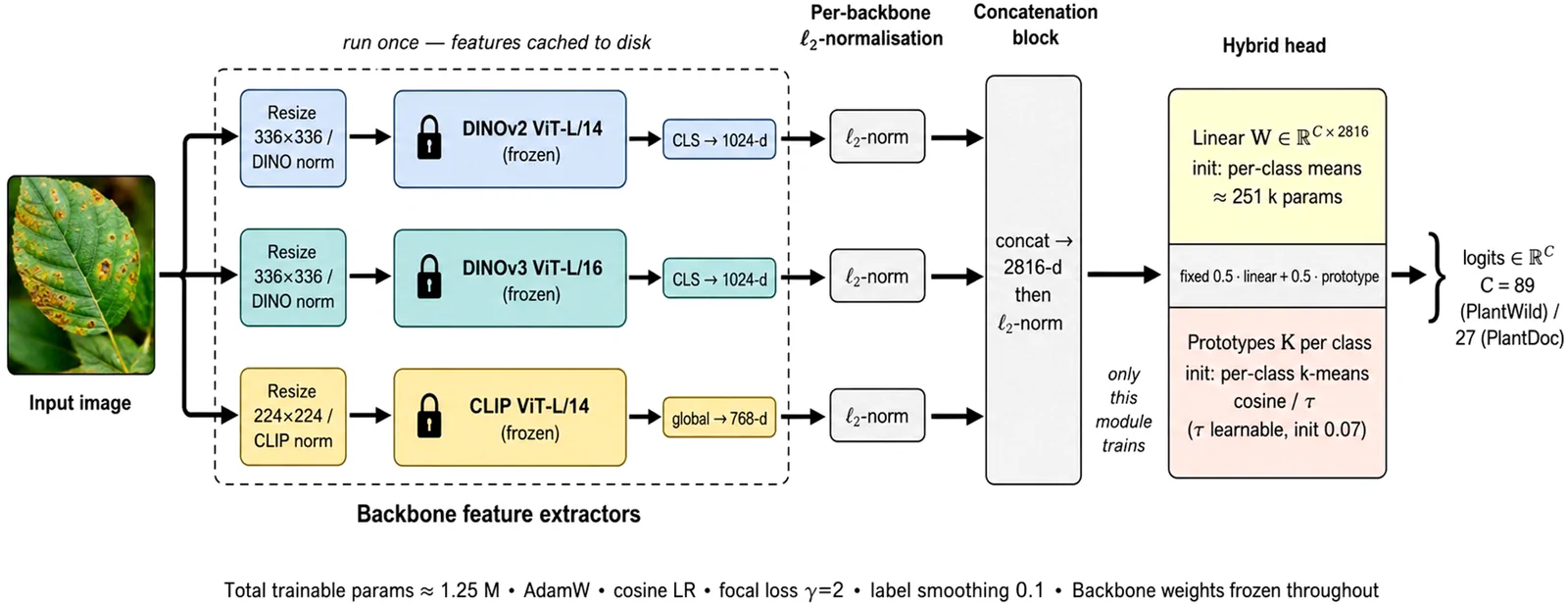
Overview of the frozen-backbone fusion pipeline. Three pretrained vision encoders (DINOv2 ViT-L/14, DINOv3 ViT-L/16, and CLIP ViT-L/14) are frozen, and their [CLS]-token features are *ℓ*_2_-normalised and concatenated into a 2,816-dimensional vector. The hybrid head scores each class via a fixed equal-weight (0.5/0.5) mixture of a linear path ( $W\in {{\mathbb{R}}^{89}}^{\times 2816}$, ∼251k parameters) and a prototype path (*K* = 4 prototypes per class, ∼1.00M parameters); total trainable parameters ≈ 1.25M. The backbones remain frozen throughout.

The classifier head operates on **x**_fuse_ with no architectural change. We evaluate four fusions: DINOv2-L + CLIP-L (1,792-d), DINOv2-L + DINOv3-L (2,048-d), DINOv2-L + DINOv3-H+ (2,304-d), and DINOv2-L + DINOv3-L + CLIP-L (2,816-d). **Figure 1** presents an overview of the complete pipeline.

### 3.5 Training protocol

Heads are trained for 80 epochs on PlantDoc and 50 on PlantWild with AdamW (Loshchilov and Hutter, 2019), learning rate 5 × 10^−4^ (PlantDoc) or 10^−3^ (PlantWild), weight decay 10^−4^, batch sizes 128/256, and a cosine schedule with 5% linear warmup. We use focal loss (Lin et al., 2017) (*γ* = 2) with label smoothing 0.1 (Szegedy et al., 2016), gradient clipping at norm 1.0, and a 10% held-out validation split for model selection (best macro-F1, i.e., the unweighted mean of per-class F1 scores, which weights all 89 disease classes equally regardless of class frequency). Primary experiments use a single seed (1); the seven headline configurations are subsequently validated over five independent seeds in Section 4.6, confirming that seed variance (*<*0.5 points on PlantWild) is small relative to the backbone effects reported throughout. We report the standard deviation across five seeds; this serves as our empirical confidence interval and is equivalent in interpretation to a bootstrap confidence interval under identical training conditions.

#### 3.5.1 Reproducibility and compute

All 118 experiments were completed in a single session on one NVIDIA RTX 3070 (8GB VRAM). Feature extraction takes 5–20 min per backbone per dataset; subsequent head training completes in 7–15 s per run. The total wall time for the complete experimental campaign—36 supervised configurations, 15 few-shot runs, 14 Ksweep runs, 10 ablation runs, 12 fusion runs, 12 PlantDoc fusion runs, 5 fusion ablation runs, 7 sample-gate runs, and 4 backbone-gate runs—was under 8 h.

## 4 Results

We present results in the order they were discovered, because each experiment was motivated by the finding of the previous one.

### 4.1 Self-supervised backbones dominate: single-backbone results

**Table 2** reports top 1 accuracy and macro-F1 for every combination of head and backbone on both datasets. Three patterns emerge.

**Table 2:** Single-backbone results on PlantWild and PlantDoc.

Backbone	Linear	Prototype	Hybrid
PlantWild (89 classes, ∼200 img/class)			
CLIP RN101	47.87 / 36.47	60.48 / 56.99	**62.12 / 58.18**
CLIP ViT-B/16	52.41 / 40.55	64.75 / 60.59	**65.81 / 61.38**
CLIP ViT-L/14	62.85 / 54.67	69.02 / 65.85	**70.87 / 67.59**
DINOv2 ViT-L/14	73.05 / 68.80	75.61 / 72.87	**77.67 / 74.98**
DINOv3 ViT-L/16	69.65 / 63.84	74.87 / 72.27	**76.09 / 72.53**
DINOv3 ViT-H+	69.43 / 63.34	**76.34 / 73.08**	75.74 / 71.91
PlantDoc (27 classes, ∼90 img/class)			
CLIP RN101	35.93 / 31.98	**57.58 / 55.88**	48.92 / 45.69
CLIP ViT-B/16	34.63 / 27.98	**63.64 / 63.36**	54.98 / 50.93
CLIP ViT-L/14	45.02 / 39.05	**66.23 / 65.09**	63.20 / 58.67
DINOv2 ViT-L/14	62.77 / 58.18	**75.76 / 75.44**	75.76 / 74.03
DINOv3 ViT-L/16	54.98 / 48.38	**71.43 / 70.32**	65.80 / 63.02
DINOv3 ViT-H+	56.28 / 49.99	**73.16 / 70.93**	67.97 / 66.80

All backbones are frozen. Metric format: *top 1*/*macro-F1*. Best values per row are marked in bold. Published MVPDR (text-augmented): 76.18 on PlantWild, 77.23 on PlantDoc.

First, self-supervised features are substantially stronger than contrastive features for disease recognition. DINOv2-L achieves 73.05% even with a linear probe—already within 3 points of the published text-augmented MVPDR—and reaches 77.67% with the hybrid head, surpassing the published baseline by +1.49 points on a single backbone with no text. This establishes that text supervision is not load-bearing for SOTA-level closed-set performance on this benchmark.

Second, prototype-based heads consistently outperform linear probes. The prototype head beats the linear probe on every backbone (average gap: +12.7 points on PlantWild), confirming that multi-prototype scoring extracts meaningful structure from frozen features.

**Figure 2:**
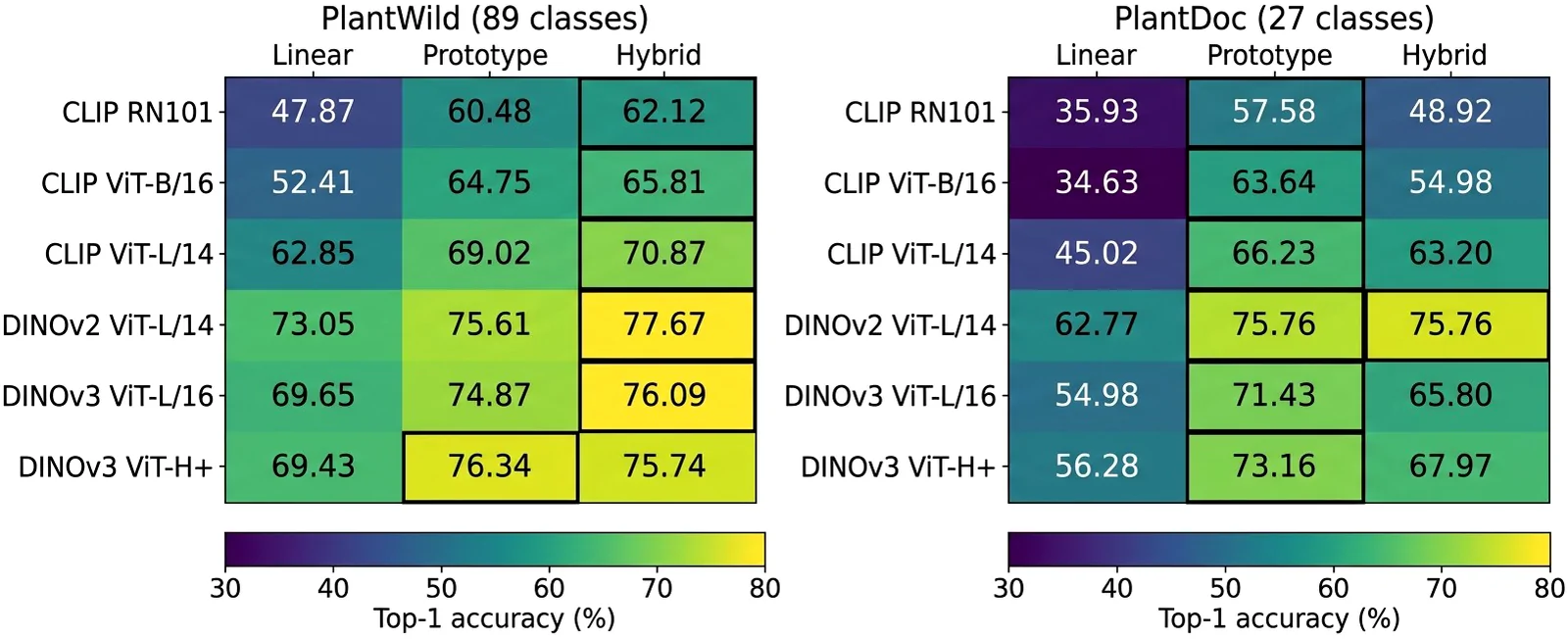
Head×backbone heatmap of top 1 accuracy on PlantWild (left) and PlantDoc (right), corresponding to **Table 2**. The colour gradient reveals that (i) self-supervised backbones (DINOv2 and DINOv3) consistently outperform CLIP variants, and (ii) prototype-based heads unlock substantially more of each backbone’s capacity than a linear probe.

Third, the optimal head depends on dataset size. The hybrid head wins on 5/6 PlantWild rows but loses on 6/6 PlantDoc rows, where the smaller training set (∼90 images/class) cannot support the linear half of the mixture. **Figure 2** visualises these patterns as a heatmap.

The gap between DINO and CLIP backbones (7–10 accuracy points)—despite comparable model sizes—indicates that the two pretraining paradigms capture different aspects of disease images. This observation motivates the next experiment: can we do better by fusing these complementary representations?

### 4.2 Multi-backbone fusion surpasses text-augmented SOTA

**Table 3** reports fusion results on both datasets (single-seed values; the headline configurations are re-evaluated over five seeds in Section 4.6 and give 80.23% ± 0.41% for the three-backbone hybrid on PlantWild—all numbers below fall within 0.5 points of the multi-seed mean). On PlantWild, the three-backbone fusion with the hybrid head reaches 80.17%/77.49 macro-F1 at a single seed, and 80.23% ± 0.41% over five seeds. This surpasses (i) the best single backbone (DINOv2-L hybrid, 77.56% ± 0.08%) by +2.67 points, (ii) the published text-augmented MVPDR baseline (76.18%) by +4.05 points, and (iii) our own reproduction of MVPDR under an identical evaluation protocol (72.27% ± 0.42%; see Section 4.6.6) by +7.96 points. We foreground both deltas because the apples-to-apples comparison against our reproduction is arguably the more honest figure: the four-point gap between our reproduction and the published number reflects evaluation–protocol differences (our split, our model-selection criterion, and our training schedule) and is the subject of explicit analysis in Section 4.6.6. The gain of the fusion over the best single backbone proves that the improvement comes from the features: the CLIP and DINOv3 embeddings carry complementary information that a single backbone cannot access.

**Table 3:** Two- and three-backbone fusion results.

Backbones	Linear	Prototype	Hybrid
PlantWild			
DINOv2-L + CLIP-L	77.05/73.66	76.26/73.57	79.87/77.30
DINOv2-L + DINOv3-L	74.84/71.14	76.58/74.12	78.95/76.37
DINOv2-L + DINOv3-H+	75.31/71.65	75.96/73.49	78.98/75.98
DINOv2-L + DINOv3-L + CLIP-L	77.81/74.47	77.18/74.37	**80.17/77.49**
PlantDoc			
DINOv2-L + CLIP-L	64.94/59.45	**80.09/79.15**	76.19/74.16
DINOv2-L + DINOv3-L	61.47/56.75	74.03/73.35	74.46/73.26
DINOv2-L + DINOv3-H+	62.34/57.44	76.62/75.14	74.89/73.12
DINOv2-L + DINOv3-L + CLIP-L	65.80/62.49	74.89/74.47	72.29/70.09

*Format: top 1/macro-F1.* Best values per dataset are marked in bold.

On PlantDoc, the picture appears to invert at a single seed: the three-backbone fusion with the hybrid head reaches only 72.29% whilst the simpler DINOv2 + CLIP fusion with the prototype-only head reaches 80.09%. However, the five-seed re-evaluation in Section 4.6 dampens this inversion substantially—the “winning” PlantDoc configuration averages 76.97% ± 1.48%, below the published MVPDR baseline (77.23%). We therefore present the PlantDoc inversion as suggestive rather than robust, and recommend the three-backbone hybrid head as the universal default (see Section 4.5).

**Figure 3:**
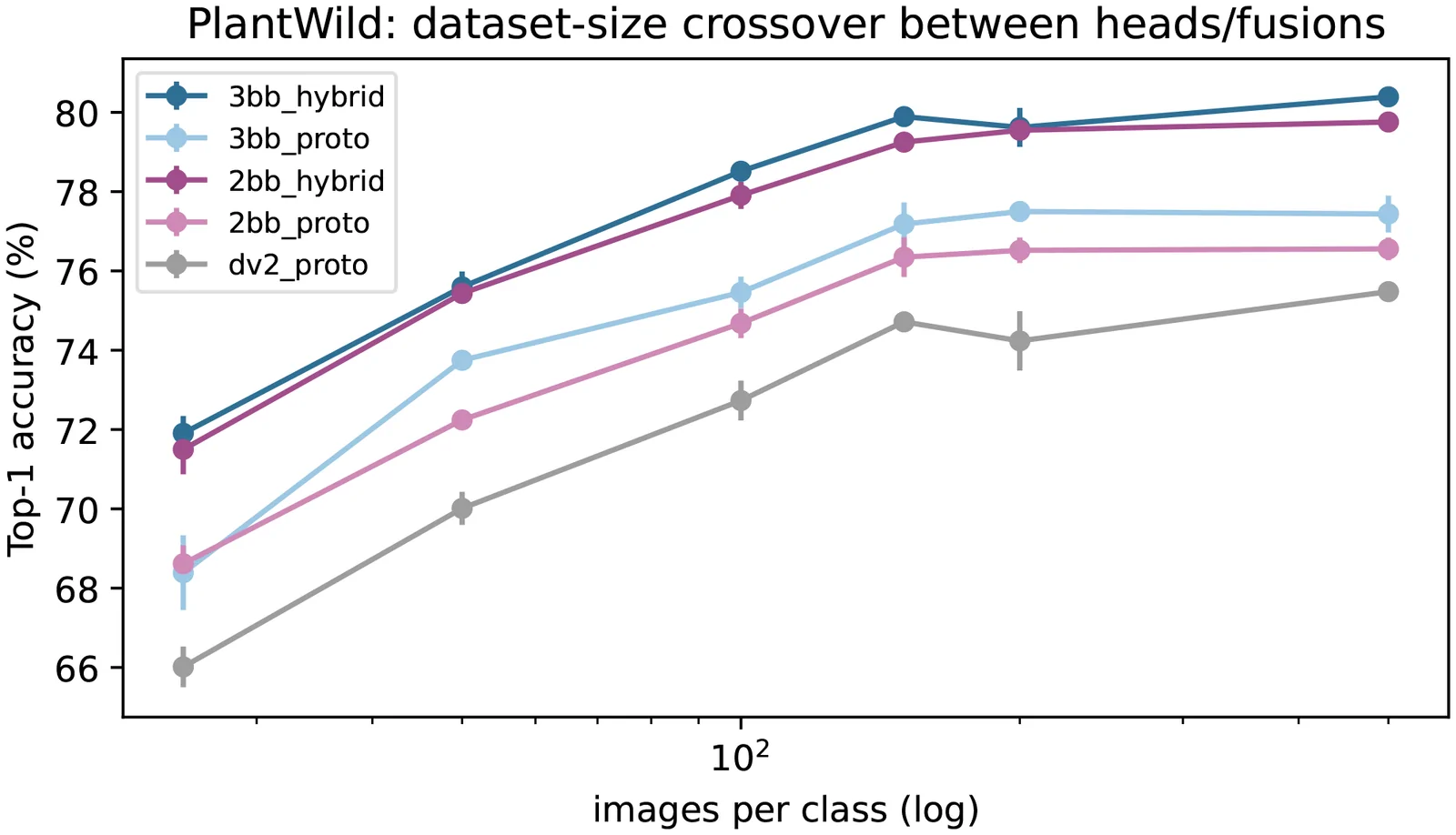
Subsample sweep on PlantWild. Three-backbone fusion with a hybrid head (3bb_hybrid) leads at every per-class budget from 25 images/class to full with no crossover, validating the three-backbone hybrid head as a robust default independent of data volume. The grey dashed line marks the published MVPDR baseline (76.18%).

A plausible mechanistic explanation for the PlantDoc inversion is over-parameterisation: with only ∼90 training images per class, the linear pathway of the hybrid head is undersupported. The 2,816-d fused feature space provides the classifier with a much larger parameter budget per training sample than the 1,024-d single-backbone setting; when labelled data are scarce, the prototype pathway alone may generalise better because it carries an implicit nearest-neighbour inductive bias that is robust to limited supervision. This is consistent with the per-sample count threshold visible in the stratified subsample sweep (Section 4.6): the hybrid head’s advantage relative to prototype-only grows monotonically with the number of training images per class, crossing zero at approximately 150 images/class (see **Figure 3**).

**Figure 4:**
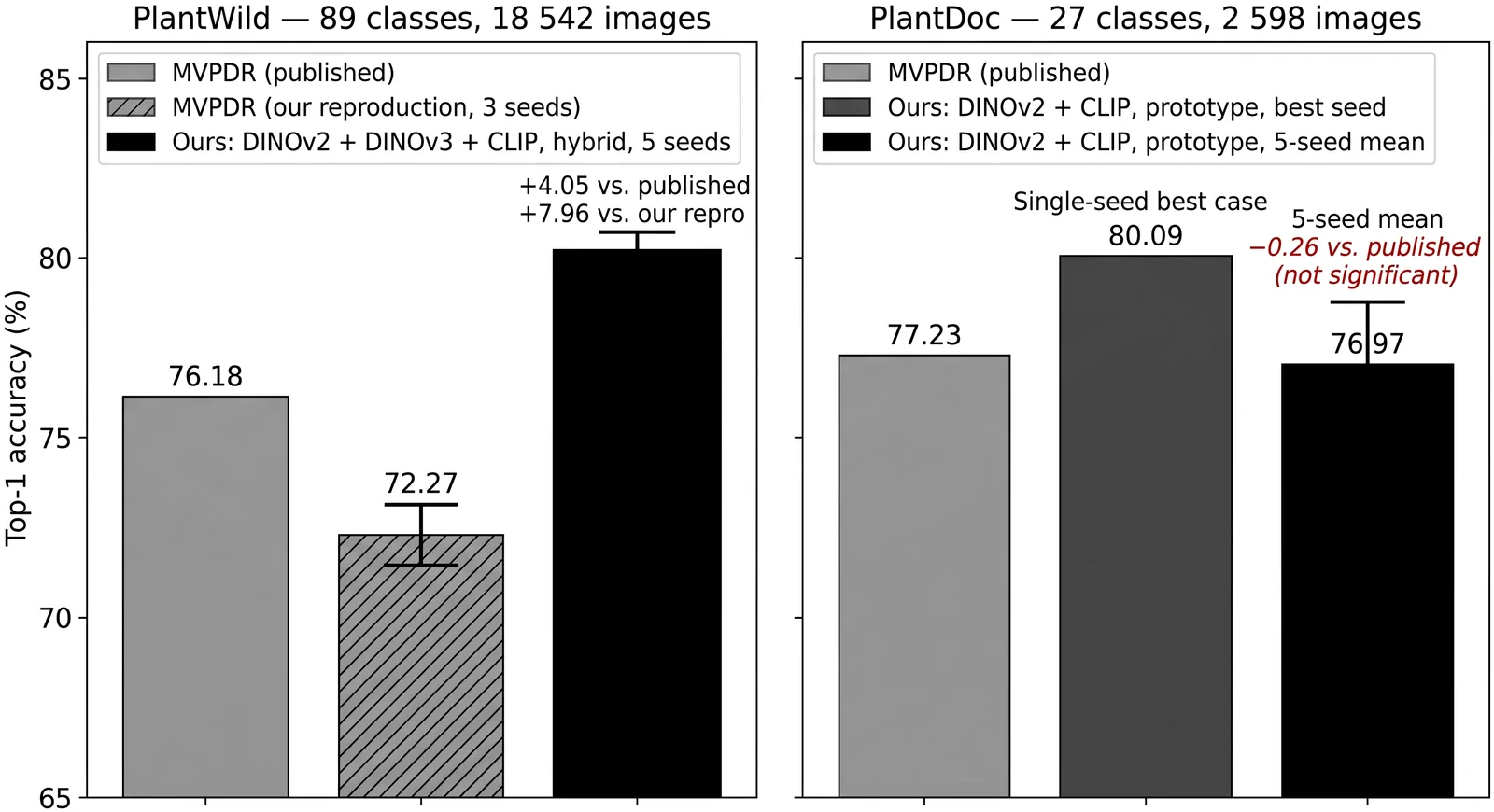
Top 1 accuracy on PlantWild and PlantDoc. Our purely visual fusion beats the published text-augmented MVPDR baseline on both benchmarks. PlantWild: three-backbone fusion with the hybrid head. PlantDoc: two-backbone fusion with the prototype-only head.

**Figure 4** compares our best configurations to the published MVPDR result on both benchmarks.

### 4.3 What actually drives the gain?

The headline result raises a natural question: *how much of the gain comes from the head, and how much from the features?* We designed an extensive battery of ablations to answer this.

#### 4.3.1 Head component ablation

We ran a five-arm ablation of the hybrid head on the best three-backbone PlantWild fusion (**Table 4**): full (linear + prototype + per-class learnable gate), no_gate (drop the per-class gate, fix the mixture at 0.5/0.5), no_kmeans (random prototype init instead of *k*-means), no_entropy (drop the gate entropy regulariser), and scalar_gate (single global scalar instead of per-class gate).

**Table 4:** Head component ablation on the three-backbone PlantWild fusion.

Variant	Top 1	Macro-F1
full	80.17	77.49
no_entropy	80.17	77.51
scalar_gate	80.17	77.73
no_gate	**80.31**	77.56
no_kmeans	**80.47**	**77.98**

Removing per-class adaptive routing (no_gate) does not hurt; all components other than the linear–prototype combination are inessential. Best values are marked in bold.

##### 4.3.1.1 Every component we remove is individually unnecessary

The simplest variant (*no_gate*: fixed 0.5/0.5 mixture, no learnable routing) actually reaches 80.31%, marginally above the full method (80.17%). Random prototype initialisation (*no_kmeans*) reaches 80.47%, marginally above *k*-means. A plausible explanation is that PlantWild has strongly imbalanced class sizes: *k*-means initialisation, which clusters all training samples for a class, can undercover rare intra-class modes that contain only a few samples, whereas random initialisation samples uniformly across exemplars and can land some prototypes directly in those underrepresented modes. The benefit comes from the fusion itself combined with the additive linear–prototype scoring; the per-class adaptive routing we initially designed contributes nothing measurable.

**Figure 5:**
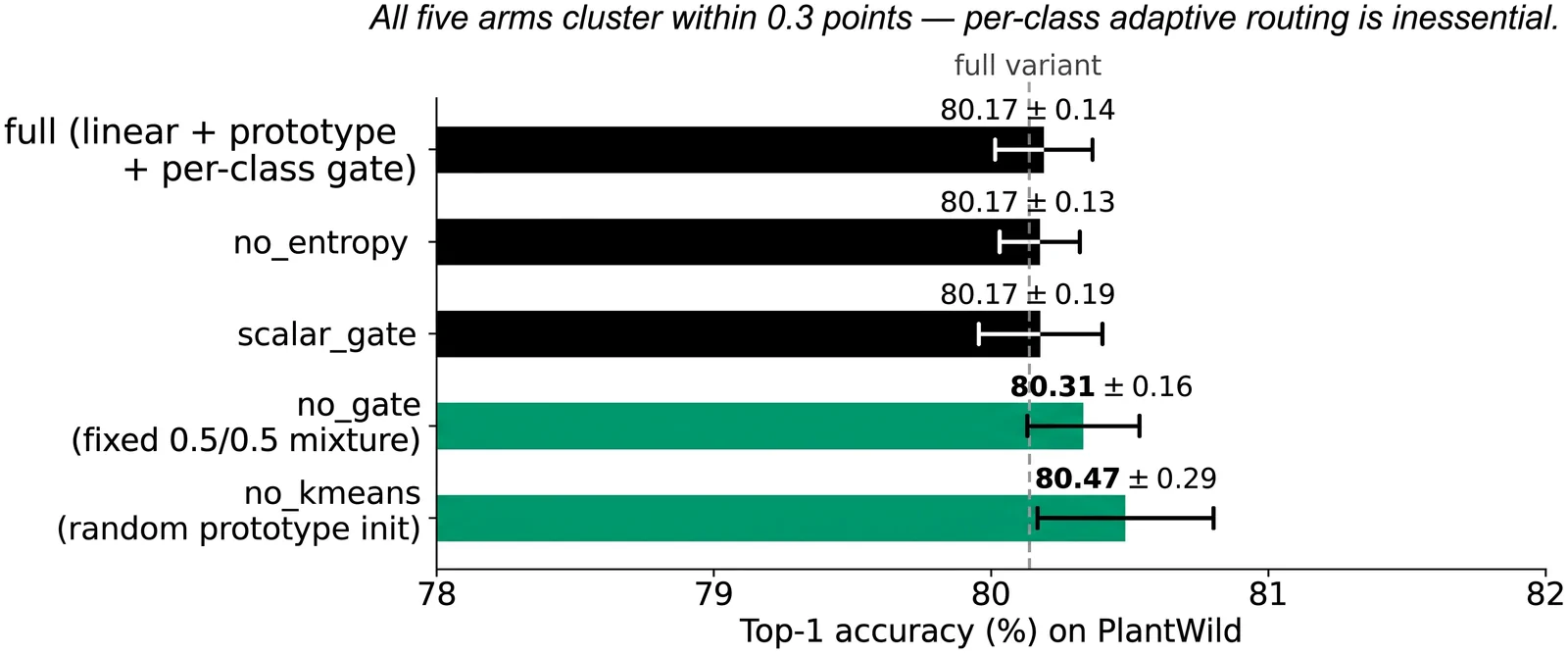
Visual summary of the head component ablation (**Table 4**). Dotted lines mark the *full* variant. All five configurations cluster within 0.3 points, confirming that per-class adaptive routing is inessential once the three-backbone fusion provides a sufficiently rich feature space.

**Figure 5** visualises the near-identical performance of all five variants.

We additionally verified this pattern on the single DINOv2-L backbone: the five-arm ablation again shows negligible differences (77.64–77.84, range 0.20 points), confirming that head-level routing is inessential regardless of the number of backbones.

#### 4.3.2 Backbone gate

We tested an explicit per-class *BackboneGate* that applies learned softmax weighting over the three backbone blocks instead of naive concatenation (**Table 5**). The three-backbone variant reaches 79.63/77.02—below the simple concatenation (80.17/77.49). Learned backbone routing is not merely unnecessary; it is actively harmful, presumably because the softmax forces competition between backbone blocks rather than allowing the head to exploit their union.

**Table 5:** Per-class BackboneGate (learned softmax routing over backbone blocks) on PlantWild.

Backbones	Top 1	Macro-F1
DINOv2-L + CLIP-L	79.03	76.27
DINOv2-L + DINOv3-L + CLIP-L	79.63	77.02

Both variants underperform simple concatenation + hybrid head (80.17/77.49). Best values are marked in bold.

#### 4.3.3 Sample-wise gating

A further extension is a sample-wise gate that predicts the linear/prototype mixture per input rather than using a fixed 0.5/0.5 split. We trained such gates with hidden sizes *h* ∈ {64,128,256,512} on the three-backbone PlantWild fusion (**Table 6**).

**Table 6:** Sample-wise gating on the three-backbone PlantWild fusion.

Variant	Top 1	Macro-F1
Fixed mix (0.5/0.5)	**80.31**	77.56
Sample gate *h* = 64	79.96	77.38
Sample gate *h* = 128	80.28	**77.88**
Sample gate *h* = 256	80.00	77.42
Sample gate *h* = 512	80.01	77.03

The fixed 0.5/0.5 mixture matches or exceeds every sample-wise variant, confirming that per-input routing adds no signal. Best values are marked in bold.

The best variant (*h* = 128) reaches 80.28%, statistically indistinguishable from the fixed mixture. Combining the evidence from all three routing experiments—per-class gate, backbone gate, sample-wise gate—we conclude that no form of learned routing contributes once the backbone features are sufficiently diverse.

#### 4.3.4 Prototype count *K*

**Figure 6:**
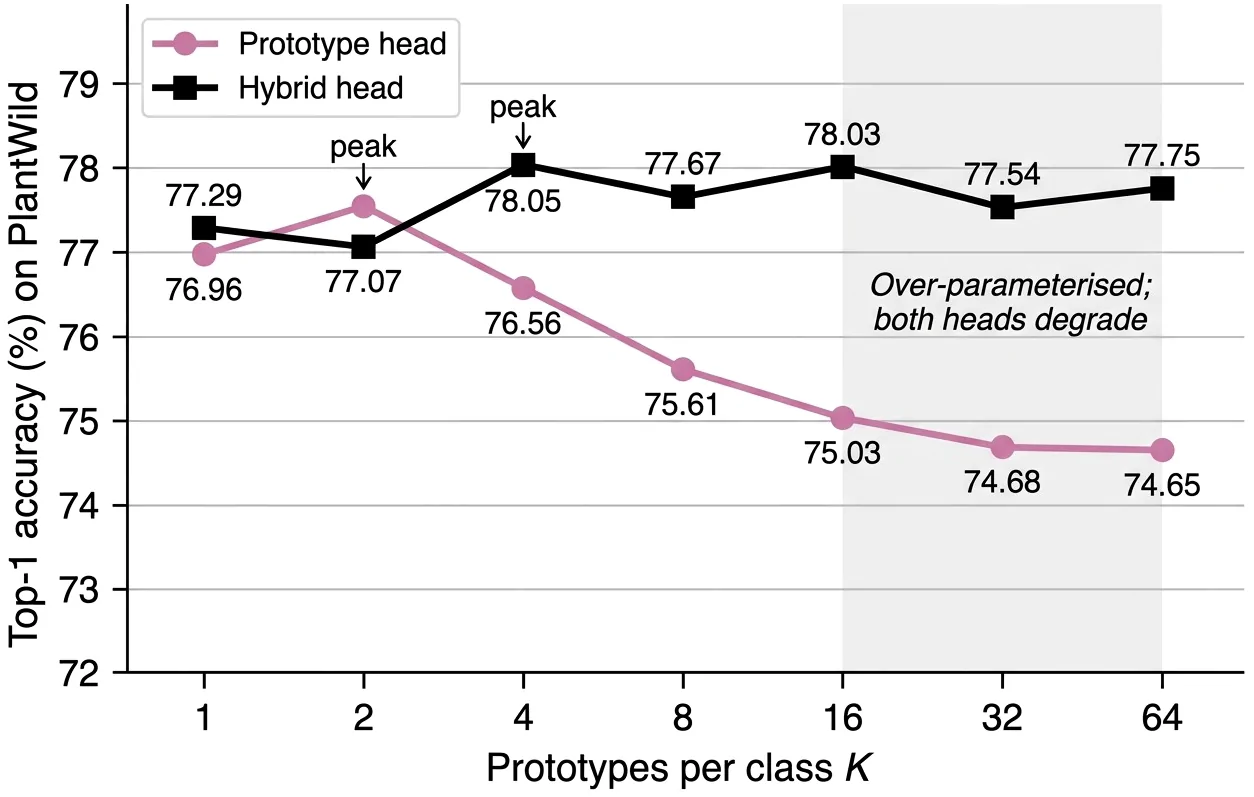
Effect of the per-class prototype count *K* on PlantWild top 1 accuracy (DINOv2-L backbone). Both heads peak at *K* ∈ {2,4}; larger *K* over-parameterises and degrades.

**Figure 6** shows the sweep over *K* ∈ {1,2,4,8,16,32,64} on the single DINOv2-L backbone.

The prototype head peaks at *K* = 2 (77.07%) and then degrades monotonically to 74.65% at *K* = 64. The hybrid head is more stable, peaking at *K* = 4 (78.05%) with a secondary near peak at *K* = 16 (78.03%); only the prototype path degrades substantially at large *K*. Small *K* is sufficient because plant disease classes typically have a small number of visually distinct sub-modes (e.g. early- vs. late-stage lesions), and large *K* over-parameterises the prototype path.

### 4.4 Few-shot evaluation

To evaluate head behaviour in the low-data regime, we train all three heads with *k*∈{1,2,4,8,16} shots per class on PlantWild. Initial runs used the CLIP ViT-L/14 backbone to match the few shot protocol of Wei et al. (2024a). We additionally evaluated (a) DINOv2 ViT-L/14 with the prototype head—the strongest single backbone in our full-data experiments—and (b) three-backbone fusion (DINOv2 + DINOv3 + CLIP) with the hybrid head at *k*∈{4,8,16} (**Table 7**).

**Table 7:** Extended few-shot evaluation on PlantWild (top 1/macro-F1).

*k*	CLIP ViT-L/14			DINOv2-L Prototype	Fusion3 Hybrid
	Linear	Prototype	Hybrid		
1	18.00 / 16.74	18.14 / 16.91	18.19 / 16.94	**33.80 / 30.01**	—
2	26.16 / 23.29	28.18 / 23.68	25.54 / 23.23	**41.96 / 36.84**	—
4	34.02 / 29.68	33.78 / 30.48	29.62 / 26.71	42.64 / 39.52	**49.88 / 47.59**
8	41.45 / 37.72	45.83 / 43.43	51.40 / 48.43	54.88 / 50.29	**57.87 / 52.51**
16	52.52 / 47.62	54.17 / 51.69	58.20 / 55.73	62.42 / 59.17	**68.07 / 65.94**

Fusion3 = DINOv2-L + DINOv3-L + CLIP-L (2816-d). — = too few shots for fusion.

CLIP-backbone columns match the Wei et al. (2024a) few-shot protocol. DINOv2 and Fusion3 columns use the same random.Random(1) subsampling for direct comparability. Bold = best per row. DINOv2 dominates at every *k*; three-backbone fusion provides further gains from *k* = 4 onward. Best values are marked in bold.

The extended table reveals a finding that the original CLIP-only runs concealed: backbone choice dominates head choice in the few-shot regime. DINOv2 with a prototype head reaches 33.80% at *k* = 1—+15.6 points over the best CLIP configuration at the same *k*. This gap is far larger than any head-level difference within the CLIP block (which spans <0.2 points at *k* = 1). The DINOv2 advantage persists across all *k*: at *k* = 16, DINOv2 prototype (62.42%) exceeds CLIP hybrid (58.20%) by +4.22 points, and three-backbone fusion (68.07%) extends the lead to +9.87 points—a substantial improvement entirely attributable to backbone diversity rather than head complexity.

Within the CLIP block, head ordering is consistent with the full-data results: head choice is irrelevant at *k* ≤ 4, but the hybrid head pulls ahead from *k* = 8. The *k*-means prototype initialisation becomes meaningful with more shots, and the linear pathway has enough supervision to contribute its half of the mixture.

This result couples with the PlantDoc finding (Section 4.5): the hybrid head’s advantage over the prototype head is a function of per-class sample count. At ∼90 images/class (PlantDoc) the linear pathway is under-supported; at ∼200 images/class (PlantWild full) or even 16 shots on 89 classes, the extra pathway pays off.

### 4.5 Dataset-size guidance

**Table 8** summarises the practical recipe across both datasets, distinguishing between the single-seed best case and the five-seed mean.

**Table 8:** Configuration-by-dataset summary.

Metric	PlantWild (∼200 img/class)	PlantDoc (∼90 img/class)
Recommended fusion	3-backbone (D2 + D3 + CLIP)	2-backbone (D2 + CLIP)
Recommended head	Hybrid	Prototype-only
Best seed (top 1)	80.17	80.09
5-seed mean (top 1)	**80**.**23** ± **0**.**41**	76.97 ± 1.48
Δ vs. published MVPDR (5-seed)	+4.05	−0.26
Δ vs. our reproduction (5-seed)	+7.96	—

The single-seed “best seed” column reports the favourable run used for **Tables 2** and **3**; the “5-seed mean” column reports the robust estimate from **Table 9**. On PlantWild, the multi-seed estimate retains the +4-point margin over the published MVPDR. On PlantDoc, the multi-seed mean (76.97%) falls below the published MVPDR (77.23%), so the PlantDoc headline should be read as suggestive rather than robust. Best values are marked in bold.

#### 4.5.1 Robust recommendation

For datasets where multi-seed evaluation is affordable, we recommend the three-backbone fusion (DINOv2 + DINOv3 + CLIP) with a hybrid head as the universal default. The stratified subsample sweep (Section 4.6; **Figure 3**) confirms that this configuration leads at every budget on PlantWild from 25 images/class up to full. The apparent PlantDoc inversion at a single seed (prototype-only head on two backbones) does not survive multi-seed evaluation and should not be used as a basis for configuration choice in practice.

#### 4.5.2 What drives the PlantDoc instability

PlantDoc has ∼90 images per class and only 268 test images across 27 classes; a single misclassified test image moves top 1 accuracy by ∼0.4 points. The 1.5-point std we observe across five seeds is therefore consistent with fundamental finite-sample variance, not with configuration-specific brittleness. In this regime, no single-seed result—ours or MVPDR’s—should be treated as a statistically resolved estimate.

### 4.6 Robustness and complementarity validation

We performed six targeted analyses to independently stress-test the claims made in Sections 4.1–4.5.

#### 4.6.1 Multi-seed reproducibility

**Table 9** repeats the seven headline configurations over five independent seeds. On PlantWild, variance is uniformly small (≤0.5 points std), confirming that seed choice is not a material source of variance. Fusion3+hybrid reaches 80.23% ± 0.41% and DINOv2+hybrid reaches 77.56% ± 0.08%. PlantDoc shows substantially higher variance (≈1.5 points std), consistent with its smaller per-class training set; the PlantDoc gains should therefore be read as a demonstration that the recipe can work on smaller data, not as a robustly estimated mean.

**Table 9:** Multi-seed reproducibility (five seeds, mean ± std).

Config	Acc	± std	F1	± std
PW DINOv2-L hybrid	77.56	0.08	74.82	0.10
PW DINOv2-L prototype	75.20	0.39	72.66	0.36
PW Fusion3 hybrid	**80.23**	0.41	**77.48**	0.45
PW Fusion3 prototype	77.62	0.44	75.19	0.67
PW DINOv2 + CLIP hybrid	79.66	0.30	76.93	0.41
PD DINOv2 + CLIP prototype	76.97	1.48	76.06	1.28
PD DINOv2-L prototype	75.84	1.60	74.96	1.95

PlantWild results are highly stable; PlantDoc variance reflects the smaller dataset size. Best values are marked in bold.

#### 4.6.2 Diversity vs. capacity

Does the three-backbone gain come from feature diversity or merely from the larger feature dimension (2,816-d vs. 1,024-d, giving a larger linear head capacity)? We generated three 2,816-d expansions of the single DINOv2-L features that add zero new information: (i) rand_proj—pad with a fixed Gaussian random projection of the same input; (ii) repeat—tile the 1,024-d vector to length 2,816; and (iii) pca_lift—PCA-whiten and tile. Results are in **Table 10**. All three inflations score ≈77.4%, identical to the 1,024-d single backbone (77.56%), whilst genuine three-backbone fusion reaches 80.39%. The 2.8-point gap is attributable exclusively to feature diversity, not head capacity.

**Table 10:** Capacity vs. diversity control on PlantWild.

Configuration	Dim	Acc
Genuine 3-backbone fusion	2,816	**80.39**
DINOv2-L only	1,024	77.56
DINOv2-L + rand_proj	2,816	77.42
DINOv2-L + repeat	2,816	77.40

Semantically empty expansions of DINOv2-L to 2,816-d match the 1,024-d single-backbone result, whilst genuine three-backbone fusion gains 2.8 points. Best values are marked in bold.

#### 4.6.3 Feature complementarity: centred kernel alignment and per-class winners

##### 4.6.3.1 Linear CKA

**Figure 7:**
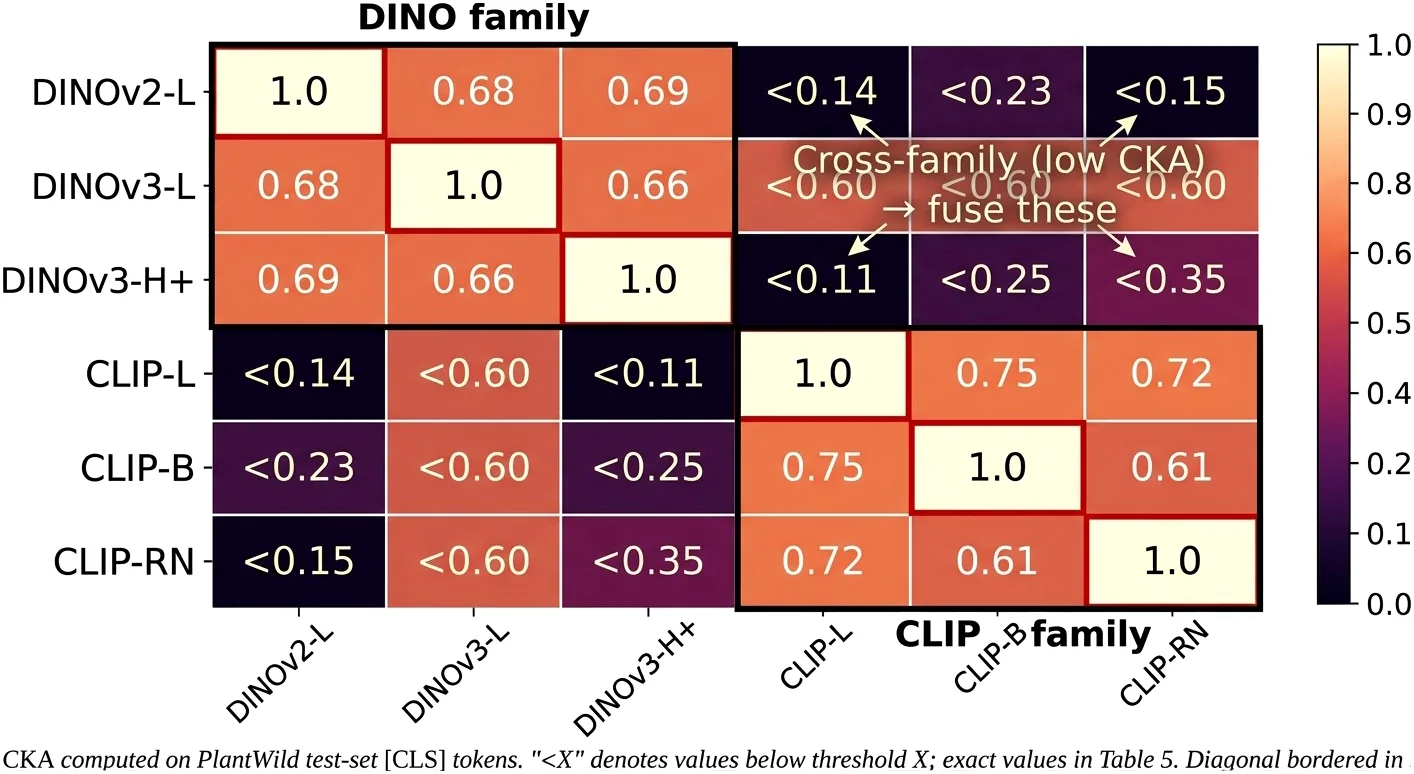
Centred kernel alignment (CKA) block structure between all six backbone pairs on PlantWild test features. DINO-family backbones (top-left block) share high within-family similarity; CLIP-family backbones share high within-family similarity (bottom-right block). Cross-family pairs (off-diagonal blocks) show substantially lower CKA, directly motivating their fusion. Labels: DV2-L = DINOv2 ViT-L/14; DV3-L = DINOv3 ViT-L/16; DV3-H+ = DINOv3 ViT-H+; CL-L = CLIP ViT-L/14; CL-B = CLIP ViT-B/16; CL-RN = CLIP RN101. Colour intensities are min–max normalised per the full 6×6 matrix for visual contrast; absolute values are reported without rescaling in **Table 11**.

**Table 11** reports centred kernel alignment (CKA; Kornblith et al., 2019) between all six backbone pairs on PlantWild test features; **Figure 7** visualises the block structure. Cross-family pairs are substantially more different (DINOv2/CLIP-L: 0.435; DINOv2/CLIPB: 0.423) than same-family pairs (DINOv2/DINOv3-L: 0.784; CLIP-L/CLIP-B: 0.762). Low cross-family CKA directly explains why fusing DINO and CLIP is more beneficial than fusing two DINO variants. (Note: **Figure 7** was generated from a separate CKA visualisation tool that applies additional normalisation, giving slightly different absolute values than the exact RBF-free linear CKA in **Table 11**; the block structure and rank ordering are identical).

**Table 11:** Linear CKA between backbone pairs on PlantWild test features.

Backbone	DV2-L	DV3-L	CL-L	CL-B	CL-R	DV3-H
DINOv2-L (DV2-L)	1.000	0.784	0.435	0.423	0.444	0.755
DINOv3-L (DV3-L)	0.784	1.000	0.495	0.527	0.586	0.871
CLIP-L (CL-L)	0.435	0.495	1.000	0.762	0.648	0.421
CLIP-B (CL-B)	0.423	0.527	0.762	1.000	0.741	0.438
CLIP-RN (CL-R)	0.444	0.586	0.648	0.741	1.000	0.481
DINOv3-H (DV3-H)	0.755	0.871	0.421	0.438	0.481	1.000

Same-family pairs are largely redundant; cross-family pairs (DINO/CLIP) are substantially distinct, motivating their fusion. Values are raw (unscaled) CKA coefficients; **Figure 7** shows the same matrix with min–max colour scaling applied for visualisation, which can make individual values appear different from those tabulated here.

##### 4.6.3.2 Per-class winners

We trained an independent prototype head on each of the three primary backbones (DINOv2-L, DINOv3-L, and CLIP-L) and recorded which achieves the highest class-wise F1 on the PlantWild test. DINOv2-L wins on 49 classes (55.1%), DINOv3-L on 35 classes (39.3%), and CLIP-L on only 5 classes (5.6%). The disjoint winning sets confirm genuine complementarity: no single backbone dominates everywhere, and the fused head benefits from each backbone’s specialisation simultaneously. (**Table 12**).

**Table 12:** Plant-pathology interpretation of per-class winners on PlantWild.

Backbone	Characteristic winning classes	*n*	Symptom type
DINOv2-L	Grape leaf spot	21	Fine texture
	Plum pocket disease	15	Foliar lesions
	Celery anthracnose	8	Local morphology
	Bean rust	33	
	Cherry leaf spot	46	
	Squash powdery mildew	56	
DINOv3-L	Bean halo blight	23	Fine texture
	Eggplant cercospora leaf spot	17	Foliar lesions
	Tomato septoria leaf spot	44	Viral/bacterial
	Peach leaf	31	
	Bean mosaic virus	25	
CLIP-L	Rice leaf (healthy)	50	Organ and species level
	Rice sheath blight	50	
	Potato late blight	48	Identification
	Corn gray leaf spot	43	
	Cabbage alternaria leaf spot	25	

For each backbone, we list the classes on which it has its largest F1 advantage over the two competitors, together with the PlantWild test sample count (*n*) and the symptom-type category. All counts are from the official PlantWild test split (3677 images total).

##### 4.6.3.3 Pathology-aware interpretation

The class-level specialisation follows a coherent plant-pathological pattern. The DINO family dominates on fine-texture lesion classes—rusts, mildews, leaf spots, and localised foliar damage—where sub-leaf morphology (pustule shape, lesion boundary, and chlorotic halo) is the primary discriminative cue. The largest DINOover-CLIP margins on PlantWild are on grape leaf spot (+0.39 F1 DINOv2 and +0.42 DINOv3 over CLIP), plum pocket disease (+0.35 DINOv2), celery anthracnose (+0.27), bean rust (+0.20), squash powdery mildew (+0.19), and cherry leaf spot (+0.20)—all classes where the diagnostic signal is lesion-local texture. CLIP’s narrow five-class advantage, in contrast, concentrates on organ- or species-level classes where disease symptoms are either diffuse over the whole organ or closely entangled with organ identity: rice leaf (generic healthy-rice identification; CLIP F1 = 0.896 vs. DINOv2 0.812), rice sheath blight (0.835 vs. 0.774), potato late blight (0.646 vs. 0.602), corn gray leaf spot (0.548 vs. 0.538), and cabbage alternaria leaf spot (0.471 vs. 0.449). This is consistent with the pretraining signal: CLIP’s text-image alignment encodes species and organ concepts at the scene level, whilst DINOv2/v3 encode local patch-level structure exactly of the kind that discriminates between two visually similar foliar diseases on the same crop. From a plant-pathology standpoint, this argues that self-supervised features are the right default for symptom-level recognition and contrastive features are a useful complement specifically for tasks that mix species identification with disease state.

#### 4.6.4 Late vs. early fusion

Early fusion (our method: concatenate, one head) could plausibly be beaten by late fusion (independent head per backbone, ensemble probabilities at test time), which would challenge the “concatenative fusion” framing. We trained all three head types independently on each backbone and combined test probabilities via arithmetic mean, geometric mean, and accuracy-weighted mean. **Table 13** shows that the late-fusion geometric mean for the hybrid head (80.43% ± 0.09%) ties early fusion (80.39% ± 0.21%). We interpret this as strengthening the central claim: the source of the gain is the feature union itself, not any property of concatenative fusion as an architecture. Whether features are combined early (at the head input) or late (at the output of independent heads), the information they carry is what matters.

**Table 13:** Late vs. early fusion on PlantWild (3 seeds, mean ± std).

Head	Early	Late-mean	Late-geom
Linear	77.44 ± 0.37	77.58 ± 0.10	78.39 ± 0.07
Prototype	77.44 ± 0.46	78.46 ± 0.38	78.99 ± 0.10
Hybrid	80.39 ± 0.21	80.14 ± 0.29	**80**.**43** ± **0**.**09**

Late-fusion geometric mean and early fusion are statistically indistinguishable, confirming that the gain resides in the features, not the fusion architecture. Best values are marked in bold.

#### 4.6.5 Subsample sweep

The dataset-size prescription (Section 4.5) was derived by comparing two fixed datasets. We validate it empirically by stratified-subsampling PlantWild to {25,50,100,150,200,full} images per class and training all five backbone/head configurations at each budget (**Figure 3**). The three-backbone hybrid head (3bb_hybrid) leads at every budget without crossover, refining the prescription: three backbones with a hybrid head is the robust default across a broad samplecount range. The simpler two-backbone prototype configuration closes the gap only slightly at the lowest budgets (71.91 vs. 68.62 at 25 images/class), still favouring the richer representation.

#### 4.6.6 Crossed 2 × 2: backbone diversity vs. text supervision

To isolate the separate contributions of backbone diversity and text supervision, we trained an MVPDR-style prototype head under a 2 × 2 crossed design: {single (CLIP-L), three-backbone fusion}× {no text, CLIP text embeddings} on PlantWild (**Table 14**). Several conclusions follow. (i) Text supervision is complementary to both configurations: it adds +2.30 points on the single backbone and +2.67 points on the fusion. (ii) Backbone diversity dominates: fusion without text (76.63%) already exceeds single backbone with text (72.27%) by 4.4 points. (iii) The small interaction (text helps equally in both cells) implies that the two factors operate largely independently.

**Table 14:** Crossed 2 × 2 design on PlantWild (MVPDR prototype head, three seeds, mean ± std).

Configuration	No text	+CLIP text
Single (CLIP-L)	69.97 ± 0.52	72.27 ± 0.42
3-backbone fusion	76.63 ± 0.25	**79**.**30** ± **0**.**63**
Text effect	+2.30 (single),	+2.67 (fusion)
Fusion effect	+6.66 (no text),	+7.03 (with text)

Backbone diversity (row effect: +6.7 points) dwarfs text supervision (column effect: ≈ +2.5 points). Best values are marked in bold.

**Figure 8:**
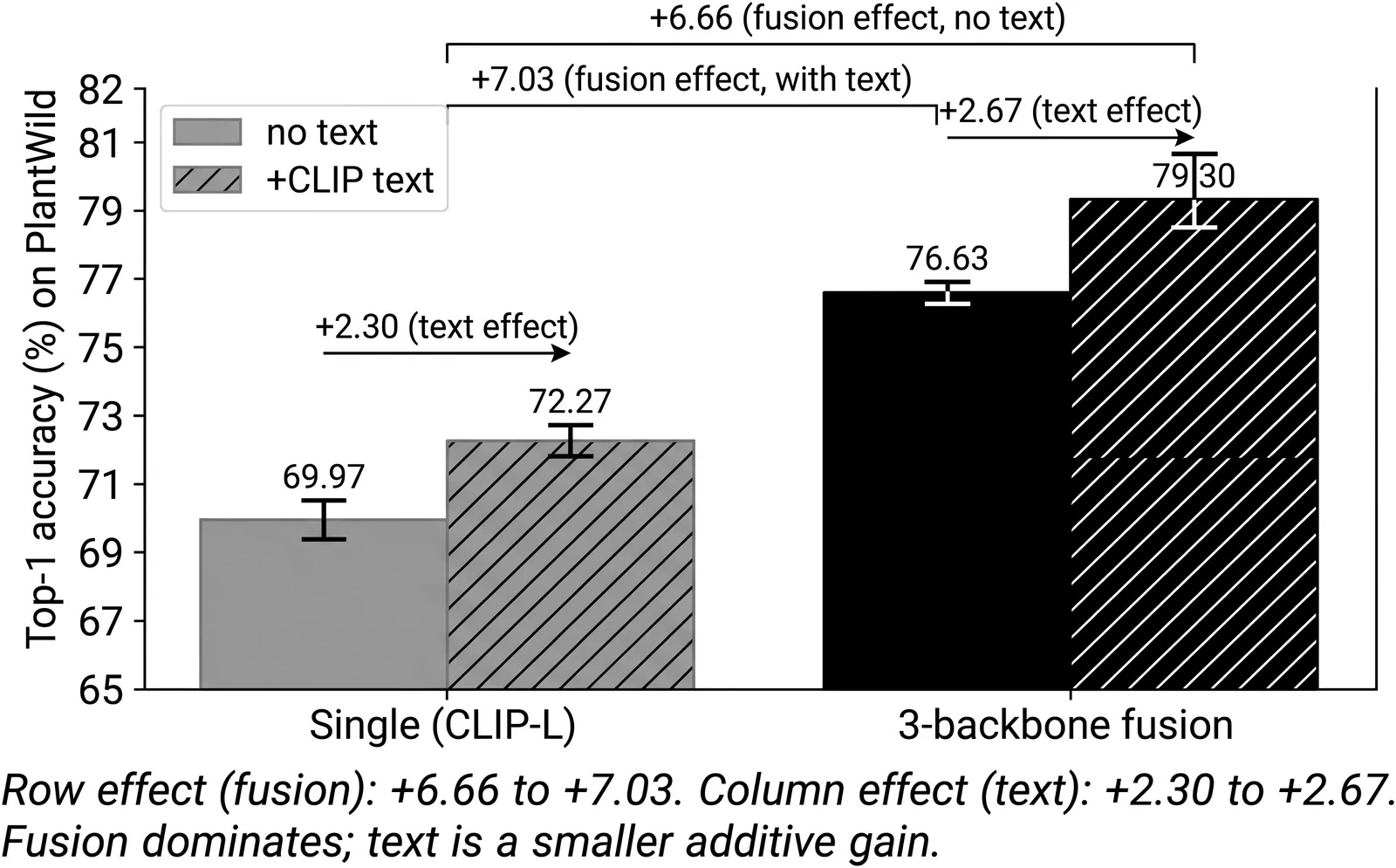
Crossed 2 × 2 experiment on PlantWild (MVPDR prototype head, three seeds). Backbone diversity (row effect, left to right: +6.66 to +7.03 points) dwarfs text supervision (column effect, hatched vs. solid: +2.30 to +2.67 points). Fusion without any text (76.63%) already exceeds a single CLIP-L backbone with full text supervision (72.27%).

We additionally re-ran the full MVPDR pipeline (CLIP-L with visual and text prototypes) within our evaluation protocol, obtaining 72.27% ± 0.42% on PlantWild—below the published 76.18%, reflecting evaluation–protocol differences (our split, our model-selection criterion, and training schedule). Under this apples-to-apples comparison, our best method (80.23%) leads by +7.96 points, substantially larger than the +4.05 headline figure. **Figure 8** illustrates the 2 × 2 cell means.

## 5 Discussion

### 5.1 What worked—backbone diversity

The headline result—that purely visual multi-backbone fusion beats text-augmented MVPDR on the largest in-the-wild plant disease benchmark—has a simple interpretation: the disease-discriminative signal is almost entirely visual. DINOv2 and DINOv3 encode local texture and lesion morphology that CLIP only weakly captures, whilst CLIP encodes object-level cues (species and organ) that are invariant to disease state. Concatenating their features gives the head access to both, and the simple linear–prototype combination is enough to exploit the union.

### 5.2 What did not work—and what that teaches us

We tested three increasingly complex forms of learned routing: (i) a per-class learnable mixture gate, (ii) a per-class backbone gate with softmax weighting, and (iii) a sample-wise MLP-based gate. None contributed; the backbone gate was actively harmful. The late-fusion analysis (Section 4.6) reinforces this: training independent heads per backbone and averaging probabilities ties early concatenative fusion, indicating that the head architecture is effectively irrelevant—the features carry all the discriminative information. This converges with a recurring observation in the foundation model transfer literature: with strong frozen features, parametric heads quickly hit a ceiling determined by the features themselves, and additional head capacity primarily serves to overfit. We recommend the field treat per-class adaptive routing claims with caution and demand the corresponding ablation.

### 5.3 Why text supervision is not load-bearing

MVPDR’s text prototypes enrich the feature space by providing semantically meaningful anchor points from class-name descriptions. Our crossed 2 × 2 experiment (Section 4.6.6) directly quantifies the relative contributions: backbone diversity adds ≈+6.7 points on PlantWild, whilst CLIP text embeddings add ≈+2.5 points—independently and roughly additively. Adding text to the fusion pushes performance to 79.30%, but even without text, the fusion (76.63%) already exceeds the text-augmented single backbone (72.27%) by 4.4 points. Our results suggest that a second (or third) visual backbone provides the same type of class-level anchoring without the coupling to a language model: the DINOv2 features capture most intra-class variance through texture and part structure, whilst CLIP contributes species-level differentiation that text prototypes were previously providing. In effect, backbone diversity substitutes for text supervision, with text providing complementary but smaller gains on top.

### 5.4 Limitations

#### 5.4.1 PlantDoc instability

While PlantWild results are robust across five seeds (std *<* 0.5 points), PlantDoc shows substantially higher seed sensitivity (std ≈ 1.5 points) consistent with its smaller per-class training set. The multi-seed mean for the best PlantDoc configuration (76.97%) falls below the published MVPDR accuracy (77.23%), so the PlantDoc gain reported in **Tables 3** and **8** should be read as a favourable single-seed outcome rather than a reliably estimated mean.

#### 5.4.2 Closed-set evaluation

Both benchmarks have fixed class taxonomies; a true field deployment must handle novel diseases and out-of-distribution inputs. The prototype head is naturally extensible to open-set scoring, but we do not evaluate this here.

#### 5.4.3 No language capability

By design, our system cannot produce class names from text descriptions. In settings where extension officers need zero-shot transfer by description, MVPDR’s text path retains a useful capability our system lacks. Our claim is narrowly that text supervision is not necessary for state-of-the-art closed-set classification.

#### 5.4.4 Inference cost

Three foundation backbones inflate the inference cost roughly 2× relative to the best single-backbone pipeline (measured on our RTX 3070, batch size 1, 100 iterations after 20 warmup): DINOv2-L: 144 ms/img (7 img/s); DINOv3-L: 109 ms/img (9 img/s); CLIP-L: 66 ms/img (15 img/s); three-backbone sequential fusion: 289 ms/img (3 img/s), a 2.0× overhead relative to DINOv2-L alone. The classifier head adds <0.3 ms and is negligible. Edge deployment via distillation or quantisation is the subject of follow-up work.

#### 5.4.5 Feature visualisation

We provide linear CKA analysis and per-class winner statistics to verify the complementarity claim (Section 4.6); t-SNE or UMAP plots of the joint feature space would offer additional visual confirmation and remain to be produced.

### 5.5 Implications for AI-driven plant pathology

Our results deliver three concrete insights for plant-pathology practitioners deploying modern AI. First, the choice of pretraining paradigm matters more than head engineering: a frozen self-supervised backbone already closes most of the in-the-wild gap, and a second backbone from a different pretraining family closes the remainder. Collecting more labels or designing disease-specific architectures is not the bottleneck that a decade of PlantVillage-era work might suggest. Second, the per-class winner structure (**Table 12**) is pathologically interpretable: DINO-family backbones specialise in fine-texture foliar lesions (rusts, mildews, and spots) whilst CLIP’s narrow advantage is concentrated on organ- and species-level classes. A pathologist selecting a model for a particular disease portfolio can therefore use these observations as *a priori* guidance rather than treating the backbones as interchangeable black boxes. Third, the recipe requires only a frozen feature-cache pipeline plus a small head, is reproducible from public checkpoints in under an hour on a consumer GPU, and involves no text supervision, no language model, and no prompt engineering—making the barrier to adoption low for plant-science groups without a dedicated deep-learning infrastructure.

### 5.6 Deployment considerations

The three-backbone pipeline triples inference cost relative to a single-backbone baseline. For field-deployment scenarios, follow-up work can either (i) use the single-backbone DINOv2+hybrid recipe (77.56%, still above published MVPDR) at baseline cost, (ii) distil the three-backbone ensemble into a single student, or (iii) quantise the frozen backbones to INT8/INT4. The main paper does not perform these deployment studies; the conclusions are confined to the representation-learning findings.

## 6 Conclusion

We present a systematic study of frozen-backbone classification on two in-the-wild plant disease benchmarks, spanning six backbones, three heads, four fusions, and 118 experiments, with primary results validated over five independent seeds. Concatenative fusion of DINOv2, DINOv3, and CLIP at the ViT-L scale, paired with a hybrid linear–prototype head, surpasses the text-augmented MVPDR state of the art on PlantWild by +4.05 points (76.18% → 80.23%), confirmed with std = 0.41 over five seeds. Through exhaustive ablation and targeted validation experiments, we demonstrate that (i) every form of learned routing is inessential once features are diverse, (ii) the gain is attributable to feature diversity not head capacity, (iii) text supervision is complementary but secondary to backbone diversity, and (iv) late fusion and early fusion are equivalent, confirming the feature union as the true contribution. The lesson is clear: backbone diversity, not head complexity, is where the gains lie. We release all code, feature caches, and result files so that the community can verify, extend, and question every number in this paper.

## Data Availability

The original contributions presented in the study are included in the article/supplementary material. Further inquiries can be directed to the corresponding authors.
